# Pattern of heart failure in Abuja, Nigeria: an echocardiographic study

**Published:** 2009-12

**Authors:** Dike B Ojji, Jacob Alfa, Samuel O Ajayi, Manmark H Mamven, Ayodele O Falase

**Affiliations:** Cardiology Unit, Department of Medicine, University of Abuja Teaching Hospital, Abuja, Nigeria; Cardiology Unit, Department of Medicine, University of Abuja Teaching Hospital, Abuja, Nigeria; Nephrology Unit, Department of Medicine, University of Abuja Teaching Hospital, Abuja, Nigeria; Nephrology Unit, Department of Medicine, University of Abuja Teaching Hospital, Abuja, Nigeria; Cardiology Unit, Department of Medicine, University College Hospital, Ibadan, Nigeria

## Abstract

**Aim:**

Despite heart failure having been identified in subjects in sub-Saharan Africa over the last 60 years, there is still a dearth of data, especially echocardiographic data on heart failure. We therefore set out to analyse the clinical and echocardiographic features of all consecutive subjects presenting with heart failure in a tertiary institution in Nigeria.

**Methods:**

Three hundred and forty subjects with heart failure, according to the guidelines of the European Society of Cardiology, were studied. Each patient had two-dimensional guided transthoracic echocardiography.

**Results:**

The mean age of the patients was 50.60 ± 15.29 years, and 50.9% of the study population were males while 49.1% were females. The commonest cause of heart failure identified was hypertension in 61.5% of the patients; 75.5% had systolic heart failure, whereas 23.5% had heart failure with preserved ejection fraction.

**Conclusions:**

Untreated hypertension has been identified as the leading cause of heart failure in Abuja, Nigeria, which is similar to that in many other parts of sub-Saharan Africa. Coronary artery disease is a rare cause of heart failure in this population group.

## Summary

With recent improvements in the control of infectious diseases and malnutrition on the one hand, and the migration to urban areas with a complete change in lifestyle on the other, cardiovascular diseases such as hypertension and cerebrovascular accident have emerged as major causes of morbidity and mortality in mostAfrican countries, including Nigeria.^1^ In most African countries, cardiovascular disease now accounts for 7–10% of all medical admissions to hospitals, with heart failure contributing a large amount of 3–7%.^2^,^3^ In spite of this, and the fact that heart failure has been identified in subjects in sub-Saharan Africa in the last 60 years,^4^ most of the published work on heart failure is based on studies carried out in developed countries, and data on heart failure in native Africans are generally lacking.^5^ Furthermore, the majority of clinical studies on heart failure in sub-Saharan Africa were carried out without the application of echocardiography.^6^-^12^

We therefore set out to analyse the clinical and echocardiographic features of all consecutive subjects presenting with heart failure at the University of Abuja Teaching Hospital from April 2006 to August 2008, in order to ascertain the pattern of heart failure in Abuja, Nigeria.

## Methods

The study was carried out at the University of Abuja Teaching Hospital, Nigeria, which is the largest tertiary health centre in the federal capital territory (FCT) of Nigeria. It receives referrals from hospitals in the FCT and neighbouring states, including Kogi, Niger, Nasarawa and Kaduna.

In this prospective, observational study, 340 eligible subjects were recruited consecutively. Subjects were eligible if they were 15 years and older, and with a confirmed diagnosis of heart failure. Heart failure was defined according to the recommendations of the European Society of Cardiology.^13^ The New York Heart Association functional class (NYHA) of subjects was also assessed.^14^

Baseline clinical and demographic characteristics of subjects were obtained using a structured questionnaire. Information obtained was gender, history of hypertension, diabetes mellitus and rheumatic fever in the past, and cigarette smoking and alcohol consumption. Subjects were weighed without shoes and in light clothing on a standard beam balance, while height was measured to the nearest centimetre using an anthropometric plane with subjects without shoes or headgear.

Body mass index was calculated using the formula weight/height^2^ and blood pressure measurements were obtained according to standard guidelines with a mercury sphygmomanometer (Accoson, London). Systolic and diastolic blood pressures were measured at Korotkoff sounds I and V respectively. Blood pressure was measured three times on the right arm after a fiveminute rest with patients in a sitting position, and the average of the three measurements was obtained. Subjects with blood pressure of 140/90 mmHg and above, or on anti-hypertensive treatment were classified as hypertensive.

## Echocardiography

M-mode and two-dimensional Doppler echocardiography with colour flow and tissue Doppler imaging were performed on all the subjects by two experienced cardiologists (using a commercially available ultrasound machine, APOGEE SSD 1600, equipped with 3.5-MHz transducer) according to the recommendations of the American Society of Echocardiography.^15^ Subjects were examined in the left lateral decubitus position using standard parasternal, short-axis and apical views. The left ventricular measurements taken included interventricular septal thickness in end-diastole (IVSDd), posterior wall thickness in end-diastole (PWTd), left ventricular internal diameter in end-diastole (LVIDd) and left ventricular internal diameter in end-systole (LVIDs). Left ventricular systolic function was calculated by Teichholz’s formula.^16^

Heart failure was classified^17^ as systolic heart failure (left ventricular ejection fraction < 50%) and heart failure with preserved ejection fraction (left ventricular ejection fraction ≥ 50%). Continuous-wave Doppler was used to interrogate the valves when there was suspicion of any valvular lesion, whereas tissue Doppler imaging was used to differentiate normal from pseudo-normal left ventricular filling.

A diagnosis of rheumatic mitral stenosis was based on the presence of thickened and/or calcified mitral leaflets and subvalvular apparatus, ‘hockey-stick’ appearance of anterior mitral valve leaflet in diastole, immobility of the posterior mitral leaflet, and narrowed ‘fish-mouth’ orifice of the mitral valve in the short axis, measurable with planimetry (valve area of ≤ 2.0 cm^2^) or Doppler echocardiography techniques.^18^

Rheumatic aortic stenosis was defined by the presence of thickened or calcified and immobile aortic valve cusps, with commissural fusion causing a narrowed orifice (valve area of ≤ 1.5 cm^2^), and almost invariably occurring with rheumatic mitral valve disease.^18^ On the other hand, rheumatic mitral and aortic regurgitations were defined by the presence of valvular regurgitation in two planes on Doppler echocardiography, and also with the following features on two-dimensional echocardiography: thickened and retracted leaflets and sub-valvular apparatus, restricted leaflet mobility, and poor coaptation of the leaflets in systole which could be worsened by dilatation of the valvular annulus.^18^ The diagnosis of degenerative valvular heart disease was also made according to the American College of Cardiology/American Heart Association 2006 guidelines for the management of patients with valvular heart disease.^18^

Idiopathic dilated cardiomyopathy was said to be present when the left ventricle was dilated (with or without dilatation of the other three cardiac chambers) with global systolic and diastolic dysfunctions in subjects with no known cause of heart failure.^19^ Peripartal cardiomyopathy was diagnosed if echocardiography revealed features of dilated cardiomyopathy (as already explained above) in the absence of a demonstrable cause or other structural heart disease, and if disease was identified for the first time within the last trimester of pregnancy or in the first five months post partum.^20^

## Statistics

Data were analysed using the Statistical Package for Social Services (SPSS) version 10.0. Baseline variables were expressed as mean ± SD. Comparison of echocardiographic parameters between subjects with systolic heart failure and those with heart failure with preserved ejection fraction was performed by the Student’s *t*-test. *P*-values < 0.05 were considered statistically significant.

## Results

Table 1 shows the clinical and demographic characteristics of the subjects studied. The mean age of the subjects was 50.60 ± 15.29 years, with 50.9% of the study population being males and 49.1% females. A minority (21.1%) of the subjects were in New York Heart Association (NYHA) functional class II, 40.7% were in functional class III and 38.2% were in class IV. Three-quarters (75.5%) of the subjects had systolic heart failure (left ventricular ejection fraction < 50%) and 23.5% had heart failure with preserved ejection fraction (left ventricular ejection fraction ≥ 50%).

**Table 1:** Demographic And Clinical Characteristics Of Subjects

*Variables*	*Values*
Total number of subjects	340
Age of subjects (years): Mean	50.60 ± 15.29
Range	15–90
Gender, *n* (%): Males	173 (50.9)
Females	167 (49.1)
NYHA class, *n* (%): NYHA II	72 (21.1)
NYHA III	138 (40.7)
NYHA IV	130 (38.2)
Systolic HF, *n* (%)	260 (75.5)
Heart failure with preserved EF, *n* (%)	80 (23.5)

NYHA = New York Heart Association classification, HF = heart failure, EF = ejection fraction.

Table 2 shows the causes of heart failure in subjects in Abuja. The commonest cause of heart failure identified was hypertension in 213 (62.6%) patients while idiopathic dilated cardiomyopathy accounted for heart failure in 37 (13.8%) cases. Rheumatic heart disease was responsible in 25 (7.4%) patients and peripartal heart disease was responsible in 11 (3.2%) cases. Other causes of heart failure, which accounted for the rest (13.0%), included degenerative valvular disease in 14 (4.1%) cases. Constrictive pericarditis, cor pulmonale, endomyocardial fibrosis, thyrotoxicosis, alcoholic cardiomyopathy and retroviral disease accounted for heart failure in eight (2.3%), six (1.8%), four (1.2%), four (1.2%), three (0.88%) and two (0.59%) patients, respectively. Other causes of heart failure identified were infective endocarditis, ventricular septal defect and use of cytotoxic drugs, each accounting for one (0.29%) each of the cases.

**Table 2:** Aetiology Of Heart Failure In Adult Subjects In Abuja, Nigeria

*Variable*	*Number*	*Percentage*
Hypertension	213	62.6
Idiopathic dilated CMP	47	13.8
Rheumatic heart disease	25	7.4
Degenerative valvular disease	14	4.1
Peripartal heart disease	11	3.2
Constrictive pericarditis	8	2.3
Cor pulmonale	6	1.8
Endomyocardial fibrosis	4	1.2
Thyrotoxicosis	4	1.2
Alcoholic cardiomyopathy	3	0.88
Retroviral disease	2	0.59
Infective endocarditis	1	0.29
Ventricular septal defect	1	0.29
Cytotoxic drug	1	0.29

Table 3 shows precipitating factors identified in 69 (25%) patients in the study population. The commonest precipitating factor identified was severely elevated blood pressure in 43 (50.6%) cases, arrhythmias (atrial fibrillation, atrial flutter and frequent ventricular premature complexes) as identified by electrocardiography in 22 (14.1%), chest infection by clinical and radiological features in 12 (14.1%) and anaemia in eight (9.4%) patients.

**Table 3:** Identified Precipitating Factors For Heart Failure In Some Of The Subjects (85)

*Variable*	*Number of subjects*	*Percentage*
Severely elevated BP	43	50.6
Arrhythmias (AF, atrial flutter, VPCS)	22	25.9
Chest infection	12	14.1
Anaemia	8	9.4

AF = atrial fibrillation, VPCS = ventricular premature complexes.

Table 4 shows the echocardiographic characteristics of subjects with systolic heart failure and those with heart failure with preserved ejection fraction. The mean end-diastolic diameter of subjects with systolic heart failure was 5.831 ± 0.98 cm compared to 4.57 ± 0.97 cm for those with heart failure with preserved ejection fraction (*p* = 0.000). The mean end-systolic diameter for subjects with systolic heart failure was 4.85 ± 1.03 cm compared to 2.83 ± 0.70 cm for subjects with heart failure with preserved ejection fraction (*p* = 0.000). Finally, 103 (30.3%) of the subject population had impaired left ventricular filling function, seven (2.1%) had pseudo-normal filling function and 135 (39.7%) had restrictive filling pattern.

**Table 4:** Echocardiographic Parameters In Study Population

*Variable*	*Systolic HF (n = 260)*	*HF with preserved EF (n = 80)*	p*-value*
Mean IVSDd (cm)	1.11 ± 0.22	1.10 ± 0.23	0.90
Mean PWDd (cm)	1.15 ± 0.32	1.08 ± 0.24	0.49
Mean EDD (cm)	6.04 ± 1.52	4.59 ± 0.98	0.000*
Mean ESD (cm)	4.85 ± 1.03	2.85 ± 0.72	0.000*
Mean EF (%)	32.9 ± 11.78	70.8 ± 9.53	0.000*
Mean DT (ms)	141.6 ± 74.7	216.8 ± 80.4	0.000*

IVSD = interventricular septal diameter in diastole, PWDd = posterior wall diameter in diastole, EDD = end-diastolic diameter, ESD = end-systolic diameter, EF = ejection fraction, DT = deceleration time, *Statistically significant.

## Discussion

This study identified untreated hypertension as the commonest cause of heart failure, degenerative valvular disease as an emerging cause of heart failure, and coronary artery disease as a rare cause of heart failure among Nigerian Africans. Untreated hypertension accounted for 62.6% of the cases studied, similar to the findings in Zaria, Nigeria.^20^ Similarly, hypertension was found to be the leading cause of heart failure in Cameroon^21^ and Ghana^22^ in 54% and 21.3% of cases, respectively.

The finding of hypertension as the leading cause of heart failure in this study further supports the fact that hypertension tends to run a more severe course with more target-organ damage in blacks compared to Caucasians. In the INTERHEART study,^23^ hypertension was found to be a strong contributor to the hazards of cardiovascular disease in black Africans. The deleterious effect of hypertension in our environment is compounded by late presentation, as many patients present to the hospital only when there are complications of hypertension, and sometimes after seeking alternative medical treatment.

Hypertension, idiopathic dilated cardiomyopathy and rheumatic heart disease were found to be the three leading causes of heart failure in this study, similar to findings in other parts of sub-Saharan Africa.^3^,^21^,^22^ In addition, idiopathic dilated cardiomyopathy accounted for heart failure in 13.2% of the subjects studied. This was lower than the report of Antony^2^ in 1980, who found idiopathic dilated cardiomyopathy to be the commonest cause of heart failure in northern savannah Nigeria in 31% of cases. A reduction in the prevalence of dilated cardiomyopathy in this study compared to the findings by Antony^2^ further supports the epidemiological transition in disease pattern being experienced by the developing nations including Nigeria, with hypertension taking a more central stage.

Degenerative valvular disease was found to be an emerging cause of heart failure in this study, being responsible for heart failure in 4.1% of cases. This interesting finding may point to the fact that a higher number of geriatric people are seeking medical care in our environment, and this may be attributable to better awareness and education on the part of the population. We also think that better nutrition and medical facilities, compared to previously, may be causing an increase in our geriatric population.

One of the cases of heart failure was secondary to the use of cytotoxic medication, which is in keeping with previous findings. 24 Three out of the four subjects with endomyocardial fibrosis were resident in the rainforest region of Nigeria and migrated to Abuja only one year before presentation, supporting the fact that endomyocardial fibrosis is a disease of the rainforest region.^6^

There was no identified case of coronary artery disease in this study (using a history of chest pain and 12-lead electrocardiography as diagnosis). This confirms the observation that coronary artery disease is not common in black Africa.^25^ This is not surprising because the prevalence of risk factors for coronary artery disease, apart from hypertension, remains relatively low in many parts of sub-Saharan Africa.^26^-^28^ However, it must be emphasised that since the diagnosis of myocardial infarction was made with only electrocardiography, with no myocardial perfusion imaging or coronary angiography performed, there may be under-estimation of the prevalence of myocardial infarction in this population.

In 85 (25%) of the subjects, some factors were identified to have precipitated a deterioration in clinical condition, leading to in-patient care. These included severely elevated blood pressure in 50.6%, arrhythmias (atrial fibrillation, atrial flutter and frequent premature ventricular complexes) in 25.9%, chest infection in 14.1% and anaemia in 9.4% of cases. In Kenya, Oyoo and Ogola3 identified inadequate therapy, arrhythmia, chest infection, anaemia and also infective endocarditis as factors associated with patient deterioration and hospital admission, while Falase *et al.*^29^ found anaemia to be a major precipitating factor in Ibadan, Nigeria.

The average age of subjects in this study was 50.60 ± 15.29 years, which is similar to findings by other workers in sub-Saharan Africa.^3^,^21^,^22^ This is unlike the developed countries where heart failure is a disease of the elderly, with an average age of incidence of 76 years.^30^,^31^ This difference was partly due to the fact that some of the major causes of heart failure in sub-Saharan Africa, such as rheumatic heart disease, idiopathic dilated cardiomyopathy and peripartum cardiomyopathy present before middle-age, and in addition, the complications of hypertension present at an earlier age compared to the Caucasian population. This early presentation of heart failure in Africa portends a bad trend as it has the potential to undermine national productivity as a consequence of the number of active life years lost by the most active workforce of the population.

A minority (23.5%) of the study population had heart failure with preserved ejection fraction. In Cameroun, Kingue *et al*.^21^ found that 10% of the subjects studied had heart failure with preserved ejection fraction, while in Europe and the United States of America 30 to 40% of their heart failure patients had heart failure with preserved ejection fraction.^32^,^33^

## Conclusion

Untreated hypertension has been identified as the leading cause of heart failure in Abuja, Nigeria, while coronary artery disease is a rare cause of heart failure in this population. Also, degenerative valvular disease was found to be gradually emerging as a significant cause of heart failure in this environment.

## References

[1] Muna WF (1993). Cardiovascular disorders in Africa.. Wld Hlth Stat Q.

[2] Anthony KK (1980). Pattern of cardiac failure in northern savanna Nigeria.. Trop Geogr Med.

[3] Oyoo GO, Ogola EN (1999). Clinical and Sociodemographic aspects of congestive heart failure patients at Kenyatta National Hospital, Nairobi.. East Africa Med J.

[4] Bedford DE, Konstam GLS (1946). Heart failure of unknown aetiology in Africans.. Br Heart J.

[5] Sliwa K, Damasceno A, Mayosi BM (2005). Epidemiology and aetiology of cardiomyopathy in Africa.. Circulation.

[6] Ladipo GO, Froude JR, Parry EH (1977). Pattern of heart disease in adults of the Nigerian Savanna: a prospective clinical study.. Afr J Med Sci.

[7] Shaper AG, Williams AW (1960). Cardiovascular disorders at an African hospital in Uganda.. Trans R Soc Trop Med Hyg.

[8] Cosnett JE (1962). Heart disease in the Zulu: especially cardiomyopathy and cardiac infarction.. Br Heart J.

[9] Abengowe CU, Das CK, Siddique AK (1980). Cardiac failure in pregnant northern Nigerian women.. Int J Gynaecol Obstet.

[10] Davidson NM, Parry EH (1978). Peripartum cardiac failure.. Q J Med.

[11] Falase AO (1979). Heart muscle disease among adult Nigerians: role of nutritional factors in its aetiology.. Eur J Cardiol.

[12] Obasohan AO, Ajuyah CO (1995). Heart failure in Nigerian hypertensive patients: the role of renal dysfunction.. Int J Cardiol.

[13] (2005). Guidelines for the treatment and diagnosis of chronic heart failure: an executive summary (update 2005).. Eur Heart J.

[14] (1964). Diseases of Heart and Blood Vessels. Nomenclature and Criteria for Diagnosis.

[15] Sahn DJ, DeMaria A, Kisslo J (1978). Recommendations regarding quantitation in M-mode echocardiography: results of a survey of echocardiographic measurements.. Circulation.

[16] Teichholz LE, Kreulen T, Herman MV (1976). Problems in echocardiographic volume determinations: echocardiographic-angiographic correlations in the presence of absence of asynergy.. Am J Cardiol.

[17] Redfield MM, Jacobsen SJ, Burnett (2003). Burden of systolic and diastolic ventricular dysfunction in the community: appreciating the scope of the heart failure epidemic.. J Am Med Assoc.

[18] Bonow RO, Carabello BA, Chatterjee K, de Leon AC, Faxon DP, Fred MD (2006). ACC/AHA 2006 practice guidelines for the management of patients with valvular heart disease: executive summary: a report of the American College of Cardiology/American Heart Association Task Force on Practice Guidelines (writing committeee to revise the 1998 Guidelines for the Management of Patients with Valvular Heart Disease).. J Am Coll Cardiol.

[19] Richardson P, McKenna W, Bristow MR (1996). Report of the 1995 World Heart Organisation/International Society and Federation of Cardiology Task Force on the Definition and Classification of Cardiomyopathies.. Circulation.

[20] Oyati IA, Danbauchi SS, Alhassan MA, Isah MS (2004). Diastolic dysfunction in persons with hypertensive heart failure.. J Natl Med Assoc.

[21] Kingue S, Dzudie A, Menanga A, Akono M, Ouankou M, Muna W (2005). A new look at the adult chronic heart failure in Africa in the age of Doppler echocardiography: experience of the Medicine Department at the Yaounde General Hospital (in French).. Ann Cardiol Angeiol (Paris).

[22] Amoah AGB, Kallen C (2000). Aetiology of heart failure as seen from a national cardiac referral centre in Africa.. Cardiology.

[23] Steyn K, Sliwa K, Hawken S, Commerford P, Onen C, Damasceno A (2005). Risk factors associated with myocardial infarction in Africa: The INTERHEART Africa study.. Circulation.

[24] Swain M, Whaley FS, Ewer MS (2003). Congestive heart failure in patients treated with doxorubicin.. Cancer.

[25] Commerford P, Mayosi B (2006). An appropriate research agenda for heart failure in Africa.. Lancet.

[26] Swaii ABM, Mclarty DG, Kitange HM (1993). Low prevalence of risk factors for coronary heart disease in rural Tanzania.. Int Epidemiol.

[27] Gebre-Yohannes A, Rahlenbeck SI (1998). Coronary heart disease risk factors among blood donors in northwest Ethiopia.. East Afr Med J.

[28] Okesina AB, Oparinde DP, Akindoyin KA (1999). Prevalence of some risk factors of coronary heart disease in rural Nigerian Population.. East Afr Med J.

[29] Falase AO, Ayeni O, Sekoni GA, Odia OJ (1983). Heart failure in Nigerian hypertensives.. Afr J Med Sci.

[30] Wenger NK (2007). The greying of cardiology: implications for management.. Heart.

[31] Goldberg RJ, Ciampa J, Lessard D (2007). Long-term survival after heart failure: a contemporary population-based perspective.. Arch intern Med.

[32] Vasan RS, Larson MG, Benjamin EJ (1999). Congestive heart failure in subjects with normal versus reduced left ventricular ejection fraction: prevalence and mortality in a population-based cohort.. J Am Coll Cardiol.

[33] Smith GL, Masoudi FA, Vaccarino V (2003). Outcomes in heart failure patients with preserved ejection fraction: mortality, readmission, and functional decline.. J Am Coll Cardiol.

